# Plantar Fasciitis Pathophysiology and the Potential Role of Mesenchymal Stem Cell-Derived Extracellular Vesicles as Therapy

**DOI:** 10.3390/biomedicines13071528

**Published:** 2025-06-23

**Authors:** Kevin Liebmann, D. Wood Kimbrough, Thomas M. Best, Dimitrios Kouroupis, Solangel Rodriguez Materon

**Affiliations:** 1 Department of Orthopaedics, UHealth Sports Medicine Institute, Miller School of Medicine, University of Miami, Miami, FL 33146, USA; kxl403@med.miami.edu (K.L.); dwk45@med.miami.edu (D.W.K.); txb440@med.miami.edu (T.M.B.); dxk504@med.miami.edu (D.K.); 2 Diabetes Research Institute & Cell Transplant Center, Miller School of Medicine, University of Miami, Miami, FL 33136, USA

**Keywords:** plantar fasciitis, biologics, mesenchymal stem/stromal cells, extracellular vesicles

## Abstract

Plantar fasciitis is a common condition characterized by inflammation and degeneration of the plantar fascia, leading to heel pain and reduced mobility. Affecting both athletic and non-athletic populations, it is a leading cause of foot-related medical visits. Conservative treatments, including rest, physical therapy, and corticosteroid injections, provide relief for most patients, but a subset experiences persistent symptoms requiring advanced therapies. Emerging biologic treatments, such as platelet-rich plasma (PRP) and mesenchymal stem/stromal cell (MSC) therapy, have demonstrated potential in promoting tissue regeneration and reducing inflammation. Recently, MSC-derived extracellular vesicles (MSC-EVs) have gained attention for their regenerative properties, offering a promising, cell-free therapeutic approach. EVs mediate tissue repair through immunomodulation, anti-inflammatory signaling, and extracellular matrix stabilization. Preclinical studies suggest that EV therapy may improve tendon and ligament healing by promoting M2 macrophage polarization, inhibiting excessive metalloproteinase activity, and enhancing vascular remodeling. This review explores the potential of MSC-EVs as an innovative, non-surgical treatment for plantar fasciitis, addressing their mechanisms of action and current evidence in musculoskeletal regeneration.

## 1. Introduction

Plantar fasciitis is a prevalent condition characterized by pain and inflammation of the plantar fascia, a thick band of tissue that runs across the bottom of the foot and connects the heel bone to the toes. Plantar fasciitis most commonly occurs between the ages of 40 and 60 and accounts for approximately 15% of foot injuries in the general population, with no significant difference between genders. While it affects both athletic and non-athletic individuals, the incidence is notably higher among runners [1,2]. With over 1 million patient visits annually in the United States, the condition poses a significant economic and personal burden [3,4]. It often causes considerable pain and disability, interfering with daily activities and overall quality of life. Risk factors for plantar fasciitis include higher BMI in non-athletes, weight-bearing activities, reduced foot muscle strength and volume, limited ankle and toe mobility, calcaneal spurs, and structural foot changes [2]. While the exact pathophysiology is still unclear, local inflammation, degeneration of the fascia, or a combination of both are thought to be the mechanisms through which plantar fasciitis develops. The degenerative mechanism of progression is described by the term plantar fasciosis; however, this term is commonly used interchangeably with plantar fasciitis to describe the condition [5].

The management of plantar fasciitis generally begins with conservative treatments, including rest, stretching exercises, ice, massage, and nonsteroidal anti-inflammatory drugs (NSAIDs). Most patients experience improvement within 12 months with these measures. Treatment then progresses to additional interventions such as physical therapy, orthotics, night splints, and corticosteroid injections [6]. Despite the effectiveness of these conservative treatments, a substantial proportion of patients experience chronic, refractory symptoms that necessitate alternative therapeutic or surgical approaches [7]. Emerging therapies such as extracorporeal shock wave therapy (ESWT) and Plasma-Rich Protein (PRP) injections have shown promise in clinical trials [8].

Mesenchymal stem/stromal cells (MSCs) are multipotent stromal cells with the ability to differentiate into various cell types, including osteoblasts, chondrocytes, and adipocytes, making them a promising tool for regenerative medicine and tissue repair [9]. More recently, MSC-derived extracellular vesicles (MSC-EVs) have gained attention for their role in intercellular communication, as they carry bioactive molecules such as proteins, lipids, and miRNAs that modulate immune responses, promote tissue regeneration, and reduce inflammation [10]. These vesicles offer a cell-free therapeutic approach with the potential to overcome some of the limitations associated with direct MSC transplantation, such as immune rejection and tumorigenicity. The purpose of this review is to explore the basis of emerging MSC-EVs therapy, as a non-surgical treatment of plantar fasciitis.

## 2. The Anatomy of the Plantar Fascia and the Pathophysiology of Plantar Fasciitis

The plantar fascia is a band of connective tissue covering the plantar surface of the foot that creates a mechanical connection between the calcaneus and the toes [11]. It is composed of medial, lateral, and central bands, and the latter constitutes the plantar fascia’s main structural and functional component [11]. The central band attaches proximally at the calcaneal tuberosity and fans as it courses distally, ultimately assuming a triangular shape [11]. At the level of the metatarsals, the fascia divides into five bands that extend digitally and insert into the plantar surfaces of the proximal phalanges [12]. Additionally, the bands make attachments to the medial and lateral intermuscular septa [11]. Inferior to the plantar fascia’s proximal enthesis is the calcaneal fat pad, which consists of pockets of adipose tissue situated between fibrous septae. This fat pad functions as a cushion for the heel while walking and protects the calcaneal tuberosity [12].

Apart from providing a mechanical connection between the toes and calcaneus, a main function of the plantar fascia is to maintain the integrity of the medial longitudinal foot arch by increasing its stiffness [11]. This function is supported by Huang et al., who found that the removal of the plantar fascia from a cadaver decreased the arch’s stiffness by 25%, which was the largest decrease associated with any ligament removal [13].

The windlass mechanism, which was coined in 1954 by Hicks et al., describes how the dorsiflexion of the toes tightens the fascia, resulting in the elevation of the arch and stabilization of the foot during gait. The increased band stretch applies a particularly high stress on the proximal enthesis of the fascia, the attachment site of the fascia to the calcaneus [11]. This “windlass” phenomenon can be exploited clinically, as passive dorsiflexion of the foot can cause pain in patients with plantar fasciitis [12]. However, a positive windlass test is not sensitive as it tends to be more commonly observed in cases involving midsubstance degeneration rather than the more typical degeneration at the calcaneal insertion [14]. Histologically, the plantar fascia has generally been thought of as a dense connective tissue and compared to both tendon and ligament. Although it may share features with both, it does not take the full identity of one or the other. Microscopically, the fascia is composed of fibrocytes organized in longitudinal rows and immersed in an extracellular matrix of collagen fibers [11]. The fibrocytes may form gap junctions with fibrocytes from adjacent rows, leading some to hypothesize a role of extracellular matrix regulation via cell–cell communication from these cells. While there is a dearth of literature describing the vascular and neuroanatomy of the plantar fascia, it is generally thought to be an avascular structure with minimal neural components. The proximal enthesis of the plantar fascia is fibrocartilaginous in nature, meaning that it consists of four distinct but contiguous portions: bone, calcified fibrocartilage, uncalcified fibrocartilage, and dense fibrous tissue of the fascia [11].

Plantar fasciitis is currently understood as a multifactorial disease that cannot be explained by one independent factor. However, there are an array of risk factors for the condition, such as abnormal biomechanics during gait, weak surrounding musculature of the foot and ankle, and increased body mass index (BMI) [6]. One mechanism through which abnormal biomechanics of gait can lead to plantar fasciitis is excessive pronation, which is common in pes planus foot types [6]. Normally, during the propulsion phase of gait, hallux dorsiflexion adequately activates the windlass mechanism of the plantar fascia. An individual with excessive pronation during gait fails to dorsiflex the hallux, placing strain on the plantar fascia [6]. Similarly, the other listed risk factors of weak musculature and increased BMI result in added stress applied to the fascia. No matter the etiology, a supraphysiological stress causes microtears, which is thought to induce an acute inflammatory response. This process constitutes the initial pathophysiological insult and response to plantar fasciitis [11].

Following the acute inflammatory response, plantar fasciitis is thought to progress via two distinct pathways that are characterized by either chronic inflammation or chronic degeneration [6]. The chronic inflammatory pathway is simply an extension and prolongation of the acute inflammatory response [6]. During the acute response, immune cells such as pro-inflammatory macrophages infiltrate the damaged tissue [15]. Prolongation of this response can cause fascial damage and subsequent heel discomfort. However, apart from chronic inflammation, plantar fasciitis can progress through chronic degeneration, which is characterized by fibroblastic proliferation and the absence of an immune component [5]. This pathway is supported by histopathological evidence, as immune infiltrates are generally absent in histology samples taken from affected patients [11]. Indeed, the main findings on such samples are mucoid, fibrinoid, and collagen degeneration with angiofibroblastic hyperplasia [11,12]. Since the term fasciitis assumes an inflammatory component, another term, fasciosis, is used to describe this variation of plantar fasciitis progression.

## 3. Clinical Presentation and Diagnosis

Patients with plantar fasciitis typically present with complaints of heel pain that is worse in the morning or after long periods of inactivity and that improves with rest [16]. Physical examination is significant for pain and tenderness to palpation of the medial calcaneal tuberosity [17]. Additional findings may include pes planus or an Achilles tendon contracture [11].

It is essential to differentiate plantar fasciitis from other common causes of heel pain, including tarsal tunnel syndrome, Achilles tendinopathy, insertional posterior tibial tendonitis, and calcaneal stress fractures. Tarsal tunnel syndrome involves nerve compression, typically producing a burning or tingling pain that differs in quality from the pain of plantar fasciitis. These patients often present with a positive Tinel’s sign and a negative windlass test [8]. Achilles tendinopathy can mimic plantar fasciitis in terms of symptom timing—pain that is worse in the morning and exacerbated by activity. However, tenderness over the Achilles tendon and posterior calcaneal tuberosity, particularly during stretching, helps distinguish it on physical exams [17]. Notably, plantar fasciitis may coexist with or result from underlying Achilles tendinopathy. Similarly, posterior tibial tendinopathy often presents with a gradual onset of medial heel pain, resembling plantar fasciitis [18]. Clinical signs such as swelling behind the medial malleolus can help with the differential [18]. Finally, calcaneal stress fractures can produce similar pain quality and location as plantar fasciitis but are uniquely identified by a positive calcaneal squeeze test, which is considered pathognomonic [19].

Most diagnoses of plantar fasciitis are made through a history and physical examination; however, in recalcitrant cases or patients’ refractory to conservative treatment, imaging can help rule out non-plantar fasciitis causes of heel pain, including heel spurs and calcaneal stress fractures. Heel spurs can exist alongside plantar fasciitis but do not cause nor are pathognomonic for the condition [12]. Ultrasound is an operator-dependent, non-invasive procedure that can assess the thickness of the plantar fascia and identify abnormalities. A normal plantar fascia should exhibit both hyperechoic and hypoechoic features, representing type I collagen and an extracellular matrix, respectively. The loss of this echogenicity (more hypoechoic features) and increased band thickness over 4 mm constitute the main findings of plantar fasciitis on ultrasound [18]. MRI is helpful in select recalcitrant cases, as it can accurately detect a thickened fascia and partial tear, determine the extent of inflammation, and identify abnormalities in the fibro–osseus junction [18].

## 4. Cellular Response in Plantar Fasciitis

Plantar fasciitis is thought to follow the processes of acute inflammation, including increased blood flow, vasodilation, and immune cell migration. Monocytes are recruited to the area and differentiate into pro-inflammatory M1 macrophages [15]. These macrophages release inflammatory cytokines that include interleukin-1 (IL-1), interleukin-6 (IL-6), tumor necrosis factor-alpha (TNF-alpha), and interleukin-12 (IL-12), which potentiate the inflammatory process, stimulate the production of acute phase proteins, and increase vascular permeability [6]. IL-1, including IL-1α and IL-1β, is a key pro-inflammatory cytokine produced mainly by macrophages that initiates the inflammatory response by upregulating genes like cyclooxygenase-2 (COX-2) and inducible nitric oxide synthase (iNOS). It also stimulates the production of other pro-inflammatory cytokines and chemokines, leading to immune cell recruitment [19]. IL-6 has both pro- and anti-inflammatory roles and is produced by macrophages and T cells in response to tissue damage. It drives acute-phase protein production in the liver, promotes B cell differentiation into plasma cells, and supports Th17 differentiation via the JAK/STAT pathway [20]. TNF-α, another key pro-inflammatory cytokine, is secreted by macrophages and T cells and facilitates leukocyte adhesion and migration by upregulating endothelial adhesion molecules. TNF-α also induces IL-1 and IL-6 production, contributing to systemic inflammatory responses [21]. Lastly, IL-12, a heterodimeric cytokine produced by dendritic cells and macrophages, is essential for Th1 differentiation, enhances natural killer (NK) cell cytotoxicity, and promotes interferon-gamma (IFN-γ) production, further amplifying immune activation [22]. If the inflammatory stimulus persists, chronic inflammation can lead to long-term damage and structural changes in the plantar fascia.

Interestingly, while inflammation might play an acute role, chronic plantar fasciitis is more thought of as a degenerative process rather than a strictly inflammatory one [15]. This condition, referred to as plantar fasciosis, is marked by fibroblastic hypertrophy, collagen disorganization, and vascular dysfunction. Fibroblasts are the primary cells responsible for the production and maintenance of the extracellular matrix (ECM) [23]. In response to tissue injury, such as repetitive mechanical stress seen in plantar fasciitis, fibroblasts can differentiate into myofibroblasts [24]. This transition is driven by mechanical cues and cytokines, particularly transforming growth factor-beta (TGF-β) [6].

Myofibroblasts are characterized by their contractile properties and their ability to produce large amounts of ECM components, including collagen. While transient myofibroblast activation is essential for normal tissue repair, persistent activation leads to pathological fibrosis. In plantar fasciitis, continuous mechanical loading can maintain myofibroblast activation, resulting in excessive ECM deposition and tissue stiffening [24].

The persistent activation of myofibroblasts in plantar fasciitis is associated with increased expression of pro-inflammatory cytokines and matrix-degrading enzymes, which further contribute to ECM degradation [25]. This pathological state disrupts the normal architecture and function of the plantar fascia, leading to pain and impaired mobility.

## 5. Current Treatment Strategies

### 5.1. Conservative Treatments

**Figure 1:**
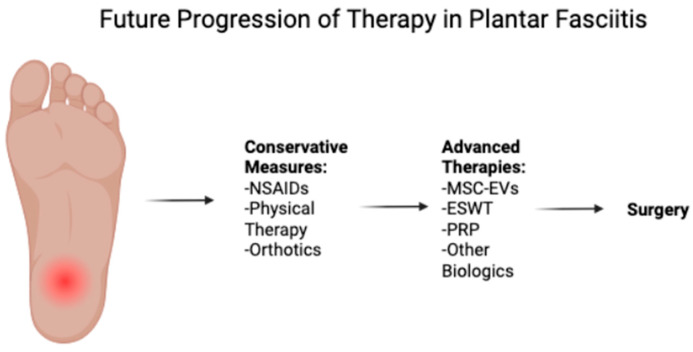
Conservative treatments remain the first-line approach for plantar fasciitis and include NSAIDs, stretching, orthotics, and physical therapy. In cases resistant to conservative measures, advanced therapies such as ESWT, MSC-EVs, and PRP injections may be utilized and prevent the necessity of surgical intervention. NSAIDS = non-steroidal anti-inflammatory drugs; ESWT = extracorporeal shockwave therapy; MSC-EVs = mesenchymal stem/stromal cell-derived extracellular vesicles; PRP = platelet-rich plasma.

Because plantar fasciitis is normally a self-limiting condition, conservative therapy constitutes the first-line therapy and takes several forms in the typical order of NSAIDs, stretching exercises for the Achilles and plantar fascia, orthotics, physical therapy, extracorporeal shockwave therapy, and corticosteroid injections (Figure 1, Table 1). Although NSAIDs are frequently given as first-line agents, a double-blinded randomized controlled trial by Donley et al. found no significant difference between the NSAID arm (celecoxib) or the placebo arm [26]. However, NSAIDs are still often used for pain reduction, particularly in the acute setting. Stretching programs are commonly employed as a combination regimen of calf stretching and plantar fascia-specific stretching. Orthotics exist in the forms of inserts, heel cups, and night splints and maintain the goal of decreasing fascial loading and improving biomechanics [12]. Some studies show that orthoses improve heel pain from baseline values [27,28]. However, these same studies show similar performance to control groups, which calls into question their benefit over simpler approaches like stretching regimens. Similarly, the evidence on the effectiveness of corticosteroid injections remains inconclusive, with no clear consensus on their ability to provide long-term pain relief. Importantly, corticosteroid injections have been associated with adverse events such as plantar fascia rupture and infection, necessitating a careful discussion with the patient [8]. There is also risk of addiction, hyperlipidemia, Cushing’s disease, and hyperglycemia. Finally, extracorporeal shock wave therapy has demonstrated success in 60–80% of cases by stimulating a healing response through controlled disruption of the fascia [8].

### 5.2. Advanced Therapies

If symptoms persist despite a conservative treatment approach, more advanced therapies, namely platelet rich-plasma (PRP) injections, stem cell injections, and surgery, can be offered to restore function and reduce pain. The procedure involves removing the affected patient’s blood, centrifuging the sample to separate cellular and non-cellular components, and injecting the platelet-rich portion locally at the affected site. Despite a plethora of studies comparing PRP injections to other treatment modalities for plantar fasciitis, the overall body of evidence remains inconclusive as to its effectiveness. Some meta-analyses have found that PRP injections may be superior to corticosteroid injections at more long term-intervals [29], whereas another meta-analysis found similar efficacies between such treatments [30]. Additionally, it is difficult to assess the true efficacy of PRP injections given variations in protocols among companies and clinicians [31].

The main surgical modalities to treat plantar fasciitis are partial and complete plantar fasciotomy, heel spur resection, and gastrocnemius recession. Both the partial and complete plantar fasciotomy can be performed via open, percutaneous, or endoscopic methods [32,33]. Some hypothesize that a complete fasciotomy can result in significantly decreased foot arch stability and a flat foot [34]. Therefore, a partial fasciotomy over a complete release is currently preferred by orthopedic surgeons. Likewise, a percutaneous fasciotomy is preferred over the heel spur resection given its lower complication rate and similar outcomes [35]. With regards to the gastrocnemius recession, a systematic review from 2022 analyzed several high-quality studies and concluded that the evidence favors its use over fasciotomies and conservative treatments [36]. While these benefits may exist, conservative therapy still remains the first-line therapy for treating plantar fasciitis.

Stem cells are undifferentiated cells that are commonly harvested from the placenta, bone marrow, or fat and can be injected into patients at the affected site. Upon injection, the cells can act to regenerate tissue or act through a paracrine mechanism to send anti-inflammatory and pro-growth signals to surrounding tissue [37]. Studies have shown that stem cells can improve pain and enhance recovery in patients refractory to conservative treatment modalities [38]. In tendinopathy, MSCs aid treatment through anti-inflammatory and analgesic effects rather than direct regeneration. Upon infusion, they reduce mononuclear cell infiltration and promote an M2 macrophage phenotype, enhancing tendon healing. This is marked by the increased expression of M2 markers (CD163, MRC1, CD204) and anti-inflammatory factors like IL-2, IL-4, prostaglandin reductase-1, and vascular endothelial growth factor (VEGF) [39,40,41]. Tendon structure relies on a well-organized extracellular matrix (ECM) and a balance between key cell populations. The two main tendon cell types are tenocytes and tendon stem/progenitor cells (TSPCs). Tenocytes are specialized, fibroblast-like cells responsible for ECM turnover and cellular metabolism. TSPCs, though less abundant, possess clonogenic, self-renewing, and multipotent properties, with the ability to differentiate into tenocytes and form tendon-like tissue in vivo [42,43]. In vitro studies suggest TSPCs have superior tenocyte differentiation potential and proliferation rates compared to bone marrow-derived MSCs, making them a promising target for tendon regeneration strategies [44,45,46]. Stem cell treatment can be limited by the potential tumorigenicity of the graft [47]. These limitations pose regulatory hurdles for stem cell treatment. However, utilizing the secretome of these cells can deliver the therapeutic effects of these cells while minimizing the risk of malignant transformation.

Other biologics include micronized dehydrated human amnion/chorion membrane (dHACM) injections. This source of growth factors and immunomodulators was found to significantly reduce pain and improve function in patients with plantar fasciitis who did not respond to conservative treatments [48]. The study included 145 subjects who were randomized to receive either dHACM injections (n = 73) or placebo (n = 72). The duration of symptoms prior to treatment was at least 1 month of failed conservative management, which included treatments such as stretching exercises, orthotics, and NSAIDs.

## 6. Extracellular Vesicles for Therapy for Plantar Fasciitis

### 6.1. What Are Extracellular Vesicles?

**Figure 2:**
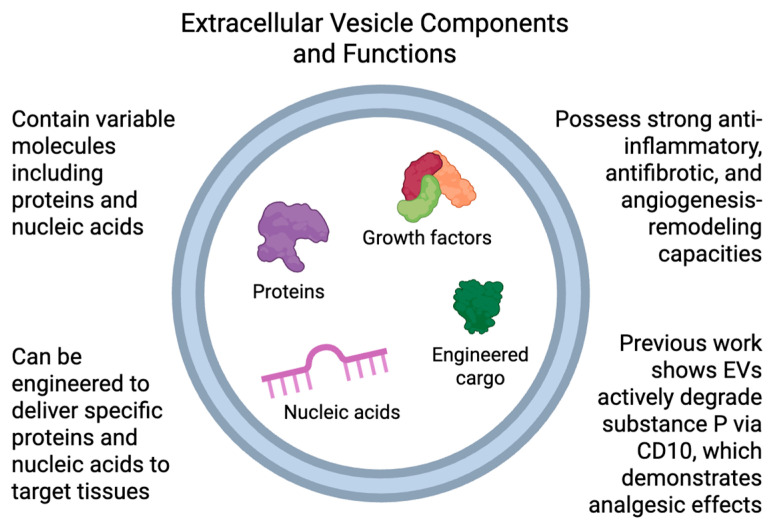
Extracellular vesicles (EVs) contain a diverse cargo, including proteins, growth factors, and nucleic acids. They can be engineered to deliver specific therapeutic molecules to target tissues, offering anti-inflammatory, antifibrotic, and pro-angiogenic effects. Engineered EVs have been shown to degrade substance P via CD10, contributing to analgesic outcomes.

EVs are membrane-bound structures secreted by MSCs. These vesicles contain a diverse array of molecules (Figure 2), including proteins and nucleic acids, which contribute to their biological functions. EVs exhibit strong anti-inflammatory, antifibrotic, and angiogenesis-remodeling properties, making them a promising tool for regenerative medicine [49].

Additionally, EVs can be engineered to deliver specific proteins and nucleic acids to target tissues, thereby enhancing their therapeutic potential. Specific molecules can be introduced into EVs through direct incubation with isolated vesicles or via electroporation, a technique that uses an electric field to temporarily increase membrane permeability, facilitating the diffusion of small molecules [49]. Beyond direct loading methods, EV content can also be modulated by altering the characteristics of the parent MSCs. By culturing MSCs under specific conditions or genetically modifying them, it is possible to influence their protein expression profiles and, consequently, the composition of the EVs they secrete [50]. Their cargo includes distinct microRNAs (miRNAs) that regulate gene expression [51,52], immune system activity [53,54], NGF/PDGF/Wnt pathways [55], protein metabolism, cell cycle regulation [56], and cellular stress responses [50]. They also contain numerous immunomodulatory proteins and growth factors that affect key signaling pathways such as PI3K-Akt, MAPK, Ras, and cytokine–cytokine receptor interactions [50]. The ability of EVs to deliver small molecules and modulate the inflammatory environment highlights their potential as promising candidates for cell-free therapies in musculoskeletal disorders.

### 6.2. Mechanisms of Action in Plantar Fasciitis

**Figure 3:**
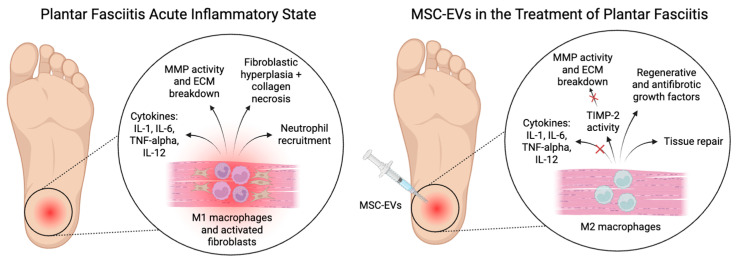
Emerging role of mesenchymal stem/stromal cell-derived extracellular vesicles (MSC-EVs) in pathophysiology and treatment of plantar fasciitis.

EVs have emerged as significant modulators of macrophage polarization and tissue repair (Figure 3). Several studies have highlighted the role of MSC-EVs in modulating macrophage polarization, accelerating the switch from M1 to M2 phenotypes, which is crucial for efficient healing [57,58,59]. In polarizing stimulated macrophages to the M2 phenotype, EVs dampen inflammation and promote tissue repair functions in macrophages.

In addition, EVs have been shown to carry tissue inhibitor of metalloproteinases-2 (TIMP-2) in their cargo. TIMP-2 can play a crucial role in inhibiting the pathogenesis of plantar fasciitis by regulating the activity of matrix metalloproteinases (MMPs). Plantar fasciitis is often associated with excessive degradation of the ECM. MMPs, particularly MMP-1 and MMP-2, degrade collagen and other structural components of the plantar fascia, leading to chronic degeneration and weakening of the tissue [60]. TIMP-2 can counteract this process by directly inhibiting MMP activity, thereby preserving the integrity of the fascia and preventing excessive tissue breakdown [61,62]. Additionally, TIMP-2 has been shown to have anti-inflammatory properties by reducing cytokine-mediated ECM degradation and fibrosis [63]. By limiting MMP activity, TIMP-2 helps maintain a balance between ECM degradation and repair, promoting proper tissue remodeling rather than pathological fibrosis or chronic degeneration [64]. This protective mechanism suggests that TIMP-2 could be a potential therapeutic target for plantar fasciitis, helping to restore tissue homeostasis and reduce chronic pain and dysfunction.

EVs also carry as cargo soluble tumor necrosis factor receptor I (sTNF-RI) [50]. This protein can play a protective role in the pathogenesis of plantar fasciitis by modulating TNF-α mediated inflammatory response. Elevated levels of TNF-α contribute to increased matrix metalloproteinase (MMP) activity, ECM degradation, and persistent pain [65,66]. sTNF-RI functions as a natural inhibitor by binding to circulating TNF-α, preventing it from interacting with its membrane-bound receptors (TNF-RI and TNF-RII). This reduces downstream pro-inflammatory signaling, including NF-κB activation, which drives the production of additional inflammatory cytokines and enzymes that contribute to tissue damage [67]. By neutralizing TNF-α, sTNF-RI helps mitigate inflammation, protect the plantar fascia from excessive degradation, and promote a more balanced tissue repair process.

EVs also contain numerous growth factors, including VEGF, fibroblast growth factor (FGF), and hepatocyte growth factor (HGF) [50]. VEGF is a key mediator of angiogenesis and vascular remodeling. VEGF plays a complex role in the treatment and progression of plantar fasciitis, particularly through its impact on inflammation and tissue repair. VEGF is a potent angiogenic factor that promotes the formation of new blood vessels, which is crucial for delivering oxygen and nutrients to the healing tissue and removing waste products. In the early stages of tendon healing, VEGF facilitates the repair process by enhancing angiogenesis, which supports the delivery of essential cells and molecules to the injury site [68]. However, the prolonged expression of VEGF can lead to excessive angiogenesis and the stimulation of MMPs [69]. Likewise, FGF can have dual effects in plantar fasciitis. While FGF promotes fascia repair, excessive FGF signaling can drive fibrosis by stimulating fibroblast activation and excessive collagen synthesis. This can lead to plantar fascia thickening and loss of elasticity, contributing to chronic pain and dysfunction [70]. Some FGF family members, like FGF-10, have been shown to modulate inflammation and reduce fibrosis, indicating a potential therapeutic role in managing plantar fasciitis [70,71]. Meanwhile, HGF is known for its regenerative and antifibrotic properties. It plays a role in suppressing excessive inflammation and fibrosis by inhibiting myofibroblast activation and promoting tissue remodeling. HGF can counteract the fibrotic effects of TGF-β, thereby reducing extracellular matrix (ECM) deposition and maintaining normal fascia elasticity [72,73]. In plantar fasciitis, HGF may promote a necessary balance to promote healing and reduce excessive collagen deposition. EVs may also affect the sensitization of pain in plantar fasciitis. A recent study showed that when stimulated rat cortical neurons are treated with infrapatellar fat pad-derived MSC-EVs (IFP-MSC EVs), there is an alteration and reduction in the neuroinflammatory profile [50]. Most notably, there is a reduction in the expression Mitogen-activated protein kinases (MAPKs). These proteins are crucial regulators of inflammation and tissue remodeling, playing a pivotal role in nociceptive signaling and the sensitization of pain pathways. The inhibition of MAPKs has been shown to exert anti-inflammatory effects in various inflammatory conditions. MSC-EVs also carry miRNA that can promote autophagy in microglia, reducing their activation and the release of pro-inflammatory cytokines, thereby alleviating neuroinflammation and pain [74].

### 6.3. Preclinical Evidence of Foot Tendinopathy Treatment with MSC-EVs

Tendinopathies, including those affecting the foot and ankle, pose significant challenges due to their chronic nature and limited regenerative capacity. Recent preclinical studies have explored EV-based therapies as a promising approach for improving tendon healing and reducing inflammation, fibrosis, and degeneration (Table 2) [75]. Although most of these studies have focused on Achilles tendon injury models, their findings have significant implications for plantar fasciitis, given the histological similarities between the plantar fascia and tendons.

Several studies have demonstrated the therapeutic potential of EVs in modulating inflammation and promoting tendon regeneration. In an enzyme-induced Achilles tendon injury model in rats, Wang et al. [59] showed that treatment with tendon stem cell-derived EVs (TSPC-EVs) significantly reduced MMP-3 expression, increased tenomodulin levels, and enhanced collagen remodeling to more closely resemble uninjured tendon architecture. Biomechanical testing further revealed improved maximum load and ultimate stress, indicating superior functional recovery [59]. Similarly, Zhang et al. (2020) found that TSPC-EVs promoted tenocyte proliferation and migration while simultaneously suppressing inflammation and apoptosis through the activation of the PI3K/AKT and MAPK/ERK1/2 pathways [76]. The activation of PI3K/AKT is essential for M2 macrophage polarization, as it not only drives anti-inflammatory gene expression but also interacts with ERK signaling to fine-tune the polarization response—ERK activation being necessary for full induction of certain M2-associated genes [77]. Together, these findings highlight the capacity of EVs to regulate tendon homeostasis and support improved healing outcomes.

In addition to TSPC EVs, adipose-derived stem cell (ASC) EVs have also been investigated for tendon repair. Shen et al. [58] reported that IFNγ-primed ASC-EVs significantly reduced inflammation by suppressing NF-κB activity and downregulating pro-inflammatory genes Il1b and IFNγ. Furthermore, treated tendons exhibited reduced gap formation and rupture rates while showing enhanced collagen deposition [58]. These findings highlight the ability of primed ASC-EVs to mitigate inflammation-driven tendon degeneration, which is also a key feature of plantar fasciitis.

Fibrotic adhesions present a major obstacle in tendon healing, often leading to compromised mobility and function. Li et al. [78] explored the use of EVs derived from hydroxycamptothecin-primed human umbilical cord stem cells (HCPT-EVs) to prevent peritendinous fibrosis in a rat Achilles tendon injury model. HCPT-EVs demonstrated superior anti-adhesion effects compared to unprimed EVs by inhibiting fibroblast proliferation, viability, and myofibroblast differentiation [78]. Mechanistically, HCPT-EVs activated the endoplasmic reticulum stress (ERS) pathway to counteract fibrosis, suggesting their potential in preventing fibrotic complications associated with chronic tendinopathies and plantar fasciitis.

Importantly, the regenerative potential of EVs varies depending on the cellular source and the biological state of the donor cells. Hayashi et al. (2022) found that bone marrow MSC-derived EVs from early-passage cells exhibited glycan patterns associated with enhanced therapeutic efficacy in Achilles tendinopathy models, whereas late-passage cells showed altered glycosylation linked to reduced regenerative potential [79]. These findings emphasize the importance of optimizing EV sources for clinical applications and suggest that careful donor selection may enhance treatment outcomes in foot and ankle tendinopathies.

In addition to MSC-EVs, dendritic cell-derived exosomes (DEXs) have emerged as another promising therapeutic avenue. Chen et al. [80] demonstrated that DEXs enhanced tendon healing in a mouse Achilles tendon rupture model by promoting collagen type I synthesis, inhibiting collagen type III, and facilitating tendon cell differentiation. Furthermore, DEXs improved the inflammatory microenvironment by shifting M1 macrophages to M2 via the PI3K/AKT pathway and reducing key inflammatory cytokines [80]. Given that chronic inflammation is a hallmark of plantar fasciitis, DEXs could provide a novel approach for managing this condition by promoting the resolution of inflammation and enhancing tissue repair.

Meanwhile, preclinical studies on PRP therapy in tendinopathy have demonstrated variable outcomes depending on timing, delivery, and combination treatments. Parafioriti et al. (2011) found that a single PRP injection temporarily improved early tendon remodeling post-Achilles rupture but showed no long-term histological benefits [81]. Rajabi et al. (2015) reported enhanced tendon healing—evidenced by increased fibroblast count and collagen deposition—when PRP was combined with aquatic exercise [82]. Chen et al. (2014) showed that PRP significantly improved histological and biomechanical healing in collagenase-induced tendinopathy, especially when combined with tendon-derived stem cells, partly via the activation of FAK/ERK1/2 signaling [83]. Yan et al. (2017) highlighted the superiority of leukocyte-poor PRP over leukocyte-rich PRP in reducing inflammation and promoting tissue repair [84]. Similarly, Lyras et al. (2010) demonstrated that PRP promoted early upregulation of TGF-β1 and reduced inflammation, contributing to improved tendon healing in a transection model [85].

Although these studies primarily focus on Achilles tendinopathy, the mechanisms underlying tendon healing and inflammation are relevant to plantar fasciitis. Like tendons, the plantar fascia undergoes degenerative changes in response to mechanical overload and chronic inflammation, leading to pain and dysfunction. The ability of EV-based therapies to suppress inflammatory cytokines, promote collagen remodeling, and improve biomechanical properties suggests their potential applicability in treating plantar fasciitis. Moreover, interventions such as HCPT-EVs, which prevent fibrosis, could be particularly beneficial in cases where plantar fasciitis progresses to fibrotic thickening, degeneration, and chronic dysfunction.

**Table 2:** Overview of preclinical studies investigating EV and PRP therapies in tendon injury models.

Author	Animal Model	Model Type	Treatment	Results
Wang et al. (2019) [59]	Rat	Enzyme-induced Achilles tendon injury	Tendon stem cell-derived EVs (TSC-EVs)	Reduced MMP-3 expression (*p* = 0.0052), induced tenomodulin expression, promoted collagen remodeling (*p* < 0.05), and improved biomechanical properties, indicating enhanced functional recovery.
Zhang et al. (2020) [76]	Rat	Achilles tendon injury	TSC-EVs	Promoted tenocyte proliferation (*p* < 0.0001) and migration (*p* < 0.0001); suppressed inflammation (*p* < 0.05) and apoptosis (*p* < 0.01) via the PI3K/AKT and MAPK/ERK1/2 signaling pathways, supporting the role of EVs in regulating tendon homeostasis.
Shen et al. (2023) [58]	Mouse	Achilles tendon injury	Interferon-γ-primed adipose-derived stem cell EVs (ASC-EVs)	Significantly reduced inflammation by suppressing NF-κB activity (*p* < 0.05) and downregulating pro-inflammatory genes Il1b and Ifng (*p* < 0.05); treated tendons exhibited reduced gap formation and rupture rates with enhanced collagen deposition (*p* < 0.05).
Li et al. (2020) [78]	Rat	Achilles tendon injury	Hydroxycamptothecin-primed EVs (HCPT-EVs) from human umbilical cord stem cells	Demonstrated superior anti-adhesion effects (*p* < 0.001) by inhibiting fibroblast proliferation (*p* < 0.001), viability (*p* < 0.001), and myofibroblast differentiation; activated the endoplasmic reticulum stress pathway to counteract fibrosis.
Hayashi et al. (2022) [79]	Rat	Achilles tendinopathy	Bone marrow MSC-derived EVs from early-passage cells	Exhibited glycan patterns associated with enhanced therapeutic efficacy, whereas late-passage cells showed altered glycosylation linked to reduced regenerative potential, emphasizing the importance of optimizing EV sources for clinical applications.
Chen et al. (2024) [80]	Mouse	Achilles tendon rupture	Dendritic cell-derived exosomes (DEXs)	Enhanced tendon healing by promoting collagen type I synthesis (*p* < 0.05), inhibiting collagen type III (*p* < 0.05), and facilitating tendon cell differentiation (*p* < 0.05); improved the inflammatory microenvironment by shifting M1 macrophages to M2 via the PI3K/AKT pathway and reducing key inflammatory cytokines (*p* < 0.05).
Parafioriti et al. 2011 [81]	Rat	Surgical Achilles tendon rupture	Single PRP injection (0.25 mL)	PRP improved tendon remodeling in the first week by enhancing tendon-like continuity, but after 2, 4, and 6 weeks, no difference was observed between PRP-treated and control groups in histology, immunostaining, or RT-PCR (*p* = 0.2). A single PRP injection was not effective for long-term healing.
Rajabi et al. (2015) [82]	Rat	Crush lesion on Achilles tendon	Aquatic activity and PRP injection	Significant increase in fibroblast number (*p* < 0.05), cellular density (*p* < 0.05), and collagen deposition (*p* < 0.05) in the combined treatment group, indicating effective tendon healing; no significant difference in tendon diameter among groups.
Chen et al. (2014) [83]	Rat	Collagenase-induced Achilles tendinopathy	Tendon-derived stem cells (TDSCs) and PRP	PRP treatment improved tendon healing, histology, and biomechanics (*p* < 0.01); PRP + TDSC combination further enhanced healing. PRP activated FAK/ERK1/2 pathways (*p* < 0.01) and tenocyte-related genes (*p* < 0.01). TDSC injection alone had little effect.
Yan et al. (2017) [84]	Rabbit	Collagenase-induced Achilles tendinopathy	Leukocyte-rich (Lr-PRP) and leukocyte-poor PRP (Lp-PRP) injections	MRI scans showed that Lp-PRP decreased signal intensity on T2 mapping compared to Lr-PRP and saline (*p* < 0.05), signifying less inflammatory edema in Lp-PRP. Lp-PRP decreased levels of IL-6 and increased levels of TIMP-1 at the lesion compared to Lr-PRP and saline (*p* < 0.05). Histology scoring showed significant improvement of the Lp-PRP group compared to Lr-PRP and saline (*p* < 0.05). Lp-PRP was found to have greater healing effects than Lr-PRP.
Lyras et al. (2010) [85]	Rabbit	Achilles tendon rupture model (transection)	Single PRP injection (1 mL)	Levels of TGF-β1 were compared between the PRP and injection groups. At weeks 1–2 post-injection, TGF-β1 levels were significantly higher in the PRP group (*p* < 0.0001). At weeks 3–4, TGF-β1 levels were significantly higher in the control group (*p* < 0.0001). The PRP group showed better healing overall with less inflammatory cells and vessels observed at 4 weeks.

### 6.4. Limitations in Clinical Translation

The field of EV research is still in its infancy, with significant limitations stemming from inconsistent terminology and methodological reporting. In an analysis of 471 EV-related clinical trials, Mizenko et al. found that two-thirds used only the term “exosomes,” underscoring the lack of standardized nomenclature. According to the 2023 guidelines from the International Society for Extracellular Vesicles, “extracellular vesicles” is the preferred generic term, while subtype classifications—such as small EVs (<200 nm) or large EVs (>200 nm)—should be based on physical or molecular properties rather than presumed cellular origin. Biogenesis-related terms like “exosome” or “ectosome” are discouraged unless the vesicle’s subcellular origin is clearly demonstrated [86]. Although clinical interest in EVs has surged—particularly since 2020 for applications such as Acute Respiratory Distress Syndrome (ARDS) using stem cell-derived EVs—only 12.1% of trials reported their isolation methods, and just 36.1% included any form of EV characterization, typically using RNA sequencing, Western blotting, or nanoparticle tracking analysis [87]. This variability in reporting and classification poses a challenge to reproducibility and translation, despite the growing momentum of EV-based diagnostics and therapeutics not only in regenerative medicine but also in cancer and vaccine research [88].

Furthermore, standardized methods for EV isolation, purification, and characterization are not yet universally accepted [89,90]. While EVs hold strong therapeutic and diagnostic promise, isolating them with high yield and purity remains challenging due to sample complexity and potential contaminants. While ultracentrifugation is cost-effective and widely used, it can compromise EV integrity. Advanced methods like density gradient centrifugation and size exclusion chromatography improve purity and structure but vary in complexity and efficiency [49].

Regulatory hurdles are further compounded by limited understanding of long-term safety, routes of administration, dosing, potential immunogenicity, and off-target effects in human subjects [91]. The therapeutic efficacy and dosing of EVs depends on the amount internalized by target cells, requiring enhanced retention in target tissues. Despite current promising findings, dosing regimens vary considerably, and the therapeutic window for EV therapy remains undefined [90]. A recent clinical trial using allogeneic umbilical cord MSC-EVs for osteoarthritis treatment has performed dose optimization, assessing clinical outcomes over a one-year period with single injections of 2 × 10^9^, 6 × 10^9^, and 2 × 10^10^ particles per dose (Trial ID NCT06431152). On this basis, as clinical trials progress, refining dosing strategies remains a critical focus to balance efficacy and safety in EV-based therapies. Also, recent advances in tissue-specific EV delivery have introduced alternative administration routes—such as intranasal, transdermal, inhalation, intramuscular injection, and in situ administration —each with distinct biodistribution and retention profiles [92]. Further, the complexity of EV trafficking at target sites and tissue may also serve as a barrier. Internalized EVs and their contents can be retained in their endosomal compartments or re-released intact by the recipient cells [93].

Finally, there is a significant translational gap for direct extrapolation to human plantar fasciitis because of anatomical and biomechanical differences between rodent and human feet. Animals that are typically utilized as disease models are not bipedal species; therefore, it is difficult to replicate the foot mechanical stress found in humans. However, studying plantar fascia stem/progenitor cells (PFSCs) might serve as an adequate in vitro model to study the pathology. In a recent study, researchers investigated the characteristics of rat PFSCs and their responses to intensive mechanical loading and IL-1β treatment, aiming to elucidate potential mechanisms underlying plantar fasciitis [25]. The study successfully isolated PFSCs and compared their properties to bone marrow MSC. Notably, both IL-1β exposure and intensive mechanical loading led to the suppression of ligament marker mRNA expression in PFSCs (Col1a1, Col3a1, Eln, Scx, Tnc, and Tnmd), accompanied by an increase in pro-inflammatory cytokines and matrix-degrading enzymes. These results suggest that inflammation and mechanical stress may alter PFSC function, contributing to extracellular matrix degeneration and impaired ligament differentiation, thereby playing a role in the pathogenesis of plantar fasciitis [25]. To address the difficulties of studying the effects EVs may have on in vivo models, PFSCs may be isolated from rat animals, exposed to pro-inflammatory environments, and treated with EVs.

## 7. Conclusions and Future Directions

EVs have demonstrated remarkable anti-inflammatory, antifibrotic, and tissue-regenerative properties through their ability to modulate macrophage polarization, regulate extracellular matrix remodeling, and suppress inflammatory cytokine signaling. The presence of bioactive molecules such as TIMP-2 and sTNF-RI within EVs further supports their role in counteracting the pathological processes underlying plantar fasciitis. These findings suggest that EV-based therapies may offer an innovative, non-surgical solution to addressing chronic plantar fasciitis, particularly in patients who have failed traditional treatments.

In conclusion, EV-based therapies represent a novel and promising frontier in the treatment of plantar fasciitis, offering a potentially transformative approach for patients with chronic, refractory symptoms. Continued research and clinical translation efforts will be crucial in realizing the full potential of this biologic therapy, ultimately improving patient outcomes and expanding the therapeutic landscape for plantar fasciitis management.

## Figures and Tables

**Table 1:** Overview of current treatments available for plantar fasciitis.

	Treatment Strategy	Advantages	Limitations and Drawbacks
**Conservative treatment**	NSAIDs	Manages pain and inflammation in the acute setting. Inexpensive and accessible.	Can cause adverse gastrointestinal and kidney side effects with long-term use. Does not treat the root cause.
	Stretching exercises	Addresses the root cause. Non-invasive.	Does not improve symptoms acutely. Normally it must be used in combination with another therapy.
	Orthotics	Improves foot biomechanics. It can be customized to the patient. Non-invasive.	Equivocal clinical data.
	Corticosteroid injections	Acutely reduces pain and inflammation. Functions locally.	Only offers short-term relief of symptoms. Equivocal clinical data. Carries several risks such as infection and plantar fascia rupture. Several injections are often required.
	Extracorporeal shock-wave therapy	Strong clinical efficacy on par with surgical intervention. Non-invasive. Quick recovery time.	Costly and generally not covered by insurance. Not universally available.
**Advanced therapies**	PRP injections	Uses autologous blood. Platelets may assist healing response.	Costly and generally not covered by insurance. Equivocal clinical data.
	Stem cell injections	Anti-inflammatory and regenerative properties. Minimally invasive compared to surgery.	Limited data. Potential for immune rejection.
	Surgery	Useful in recalcitrant cases. Different options to meet individual needs. Addresses the root problem.	Invasive with high risk profile. Long recovery period.

## Data Availability

The original contributions presented in the study are included in the article; further inquiries can be directed to the corresponding author.
